# Comparison of *TCF4* repeat expansion length in corneal endothelium and leukocytes of patients with Fuchs endothelial corneal dystrophy

**DOI:** 10.1371/journal.pone.0260837

**Published:** 2021-12-02

**Authors:** Eric D. Wieben, Ross A. Aleff, Tommy A. Rinkoski, Keith H. Baratz, Shubham Basu, Sanjay V. Patel, Leo J. Maguire, Michael P. Fautsch

**Affiliations:** 1 Department of Biochemistry and Molecular Biology, Mayo Clinic, Rochester, Minnesota, United states of America; 2 Department of Ophthalmology, Mayo Clinic, Rochester, Minnesota, United states of America; 3 Division of Biostatistics and Bioinformatics and Department of Health Sciences Research, Mayo Clinic, Rochester, Minnesota, United States of America; University of Florida, UNITED STATES

## Abstract

Expansion of CTG trinucleotide repeats (TNR) in the transcription factor 4 *(TCF4)* gene is highly associated with Fuchs Endothelial Corneal Dystrophy (FECD). Due to limitations in the availability of DNA from diseased corneal endothelium, sizing of CTG repeats in FECD patients has typically been determined using DNA samples isolated from peripheral blood leukocytes. However, it is non-feasible to extract enough DNA from surgically isolated FECD corneal endothelial tissue to determine repeat length based on current technology. To circumvent this issue, total RNA was isolated from FECD corneal endothelium and sequenced using long-read sequencing. Southern blotting of DNA samples isolated from primary cultures of corneal endothelium from these same affected individuals was also assessed. Both long read sequencing and Southern blot analysis showed significantly longer CTG TNR expansion (>1000 repeats) in the corneal endothelium from FECD patients than those characterized in leukocytes from the same individuals (<90 repeats). Our findings suggest that the *TCF4* CTG repeat expansions in the FECD corneal endothelium are much longer than those found in leukocytes.

## Introduction

Fuchs Endothelial Corneal Dystrophy (FECD) is a late onset eye disease associated with an expanded trinucleotide repeat (TNR) in the *TCF4* gene and a lack of other systemic findings. Most subjects without FECD have between 12 and 40 repeats of a CTG sequence in the third intron of *TCF4*. In 77.7% of FECD cases in the United States, the CTG repeat sequence measured in peripheral blood leukocytes contains 50 to 3000 repeats (150 to 9000 bp) [1]. Our prior work in corneal tissue established that FECD associated with CTG repeat expansion in *TCF4* (identified in peripheral blood leukocytes) leads to numerous changes in the transcriptome of FECD corneal endothelial cells, including hundreds of alterations in RNA splicing [2–4]. This work also revealed that intron sequences upstream of the CTG repeats in *TCF4* accumulate in corneal endothelial tissue obtained from FECD patients with repeat expansions. However, the ability to identify the repeat length in corneal endothelial cells is not feasible due to the inability to isolate sufficient amounts of DNA from FECD corneal surgical samples. Likewise, the use of short read sequencing of RNA has not been able to reliably determine the size and extent of transcription of the *TCF4* repeat region in corneal endothelium.

In other trinucleotide repeat-associated diseases such as myotonic dystrophy [5, 6], Friedreich’s ataxia [7], and amyotrophic lateral sclerosis/fronto-temporal dementia [8], the lengths of repeat expansion in affected tissues are significantly longer than those measured in leukocyte DNA. We hypothesized a similar process for FECD in which longer *TCF4* TNR expansion in corneal endothelial cells would associate with tissue specificity of the disease. However, the CTG repeat length has not been directly assessed in the corneal endothelium of FECD patients. The ability to use DNA as a template for repeat-length determination is ideal. However, the corneal endothelium obtained from a single, pauci-cellular surgical specimen limits the ability to extract the necessary amount of DNA required for determination of repeat length with current technology. Given that RNA-based technologies are more sensitive, RNA can be used as a surrogate to DNA given that corresponding data is available (e.g., primary cell lines). We utilized PacBio long read sequencing of mRNA (referred to as “Iso-Seq”) [9] to investigate the transcriptome of corneal endothelial cells isolated from FECD patients and controls. Using surgical samples and corresponding blood obtained from the same patients, we identified *TCF4* CTG repeat expansions longer in FECD corneal endothelial RNA than in leukocyte DNA. Analysis of repeat length in genomic DNA from primary cultures of corneal endothelial cells also revealed DNA expansions comparable to those found in corneal endothelial tissue from the same individuals.

## Methods

### Patient selection

The use of human tissue for research was in compliance with the Mayo Clinic Institutional Review Board (IRB #06–007210) and followed the tenets of the Declaration of Helsinki. Patients with advanced FECD and control patients were enrolled into the Mayo Clinic Hereditary Eye Disease Study after written informed consent and prior to endothelial keratoplasty. Descemet membrane surgical specimens from patients with non-FECD corneal edema were chosen as controls as opposed to eye bank tissue because FECD could more assuredly be ruled out based on the examination of the involved and contralateral eye, medical record review, and the absence of expansion in leukocyte DNA. At the time of surgery, the stripped Descemet membrane/corneal endothelium complex was immediately transferred to RNAlater-ICE (Invitrogen, Thermo Fisher Scientific, Waltham, MA) or Optisol GS (Bausch and Lomb, Bridgewater, NJ). Phlebotomy was performed to obtain leukocyte DNA.

### DNA isolation and short tandem repeat analysis

Leukocyte-derived DNA was extracted using AutoGen FlexiGene (Qiagen, (Valencia, CA). CTG repeats were PCR amplified by incubating 100 ng of genomic DNA with 10 pmoles of oligonucleotide primers specific for *TCF4* (5-TCF-Fuchs: 5’- CAGATGAGTTTGGTGTAAGATG-3’; 3-TCF-Fuchs 1: 5’-ACAAGCAGAAAGGGGGCTGCAA-3’) with Invitrogen Platinum PCR Super Mix High Fidelity (Carlsbad, CA) as previously described [10]. The PCR program was as follows: Hot Start 95°C, 6 min, 1 cycle; 95°C 1 min., 62°C 1 min., 68°C 3 min., 35 cycles; 68°C, 7 min., 1 cycle; and a 4°C hold.

For short tandem repeat analysis, a 5’ FAM primer (5-FAM-TCF-Fuchs– 5’-CAGATGAGTTTGGTGTAAGATG-3’) was used instead of 5-TCF-Fuchs and PCR was performed as described above. After PCR amplification, short tandem repeat analysis was performed (GeneWiz Corporation, South Plainfield, NJ).

### RNA isolation and sequencing

Total RNA was isolated from corneal endothelial tissue using the RNAeasy Mini QIAcube Kit (Qiagen, Valencia, CA). Samples were prepared using the library preparation protocol for sequencing as described in the Iso-Seq Express Template Preparation protocol (Pacific Biosciences, Menlo Park, CA) found in the Procedure & Checklist–Iso-Seq Express Template Preparation for Sequel and Sequel II Systems (Pacific Biosciences, Menlo Park, CA). This protocol can be found at “https://www.pacb.com/wp-content/uploads/Procedure-Checklist-Iso-Seq-Express-Template-Preparation-for-Sequel-and-Sequel-II-Systems.pdf.”. Each sample was sequenced in a single SMRTCell on the Pacific Biosciences Sequel II instrument, using 30 hour movies. Corresponding data can be found in the National Center for Biotechnology Information (NCBI) BioSample database under accession numbers SAMN21432327, SAMN21432328, SAMN21432329, SAMN21432330, SAMN21432331, SAMN21432332.

### Data analysis

Iso-Seq data were processed through the Pacific Biosciences Iso-Seq (v8) workflow, using default parameters. The output from this pipeline was processed through SQANTI2 [11], which guides quality control, classification of transcripts, filtering, and provides transcript open reading frame prediction.

To analyze transcribed expanded repeats, we processed individual full length non-concatemer (FLNC) sequences (herein referred to as reads) from the routine pipeline. These reads were aligned to hg19 using pbmm2 with min-concordance-perc = 95.0, min-length = 40 and the Iso-Seq preset. Individual reads including the CAG repeat were collected and the length of the repeat sequence was extracted from the sequence.

TCF4 isoforms identified by SQANTI2 were collected from the “sqanti2_classification.filtered_lite_classification.txt” file for each sample. This file provided the number of transcripts seen for each isoform identified. Each RNA isoform was associated with the sequence of the protein it coded for (taken from the collapse_isoforms.renamed_corrected.faa file). All transcripts coding for the same translation start site were grouped and quantitated. Each group of transcripts was then labeled according to the TCF4A-R start site alignment scheme used by Sepp et al. [12] and Sirp et al [13].

### Human corneal endothelial cell (HCEC) culture

Primary cultures of normal HCECs were prepared from Descemet membrane dissected from human donor eyes or from FECD patients collected at the time of endothelial keratoplasty. Tissue was stored in Optisol GS (Bausch & Lomb, Bridgewater, NJ) for ≤3 days. Following incubation, tissue was placed in Opti-MEM (Gibco, Waltham MA) with 8% fetal bovine serum (FBS; Gibco) overnight at 37°C, cells were dissociated from the tissue with 0.02% EDTA (Sigma, St Louis, MO) in PBS for 60 min at 37°C, and plated into a single well of a 6-well collagen IV-coated plate (Corning, Tewksbury, MA) containing Joyce’s media [Opti-MEM, 8% FBS, 200 mg/ml CaCl2 (Sigma), 0.08% Chondroitin sulfate (Sigma), 20 μg/ml ascorbic acid (Sigma), bovine pituitary extract 100 μg/ml (Gibco), 5ng/ml EGF (Millipore, St. Louis, MO), 50 μg/mL gentamicin (Sigma), and 1X antibiotic/antimycotic solution (Invitrogen, Waltham, MA)] [14]. HCECs were allowed to proliferate for 1–4 weeks with media changes every 3–4 days.

### Southern blot

Genomic DNA was isolated from confluent HCECs using Puregene spin kit (QIAGEN, Hilden, Germany). DNA (5 μg leukocyte DNA; 5 μg or 20 μg primary human corneal endothelial cell (HCEC) line DNA) was digested with EcoRI (New England Biolabs, Ipswitch, MA) and separated on an 0.8% agarose gel (Lonza, Basel, Switzerland) at 40V overnight. On completion of electrophoresis, gel was incubated in denaturation solution (0.5M NaOH and 1.5M NaCl) for 20 minutes at room temperature, washed in neutralization buffer (1.5M NaCl, 0.5M Tris-HCl, pH 7.5) twice for 20 minutes each, and DNA was transferred overnight to a nylon membrane (Roche, Basel, Switzerland). Membrane was UV crosslinked (Stratalinker, Stratagene, San Diego, CA) prior to hybridization with a 392 bp ^32^P-labeled DNA probe designed against unique *TCF4* sequence immediately adjacent to the CTG repeat. Following overnight incubation at 45°C, membrane was washed once in 2XSSC/0.1% SDS at room temperature for 20 minutes, twice in 2XSSC/0.1% SDS at 60°C for 20 minutes each, and once in 0.2XSSC/1% SDS at 60°C for 20 minutes. Membrane was imaged using a GE Typhoon phosphorimager (Boston, MA).

## Results

### Patient demographics

Clinical and demographic details for patients whose corneal endothelial samples were selected for Iso-Seq are shown in Table 1. All subjects were Caucasian. Each of the three FECD patients [FECD(1–3)] had bilateral disease. Descemet membrane surgical specimens from patients with non-FECD corneal edema were chosen as controls as opposed to eye bank tissue because FECD could more assuredly be ruled out based on the examination of the involved and contralateral eye, medical record review, and the absence of expansion in leukocyte DNA.

**Table 1:** Patient demographics.

Patient	Sex	Age	Ophthalmic Condition
Iso-Seq			
FECD(1)	M	58	FECD
FECD(2)	F	61	FECD
FECD(3)	M	67	FECD
Cont(1)	M	79	Pseudophakic corneal edema, glaucoma, Baerveldt implant
Cont(2)	F	78	Pseudophakic corneal edema, glaucoma, pseudoexfoliation, trabeculectomy
Cont(3)	F	74	Pseudophakic corneal edema, glaucoma, trabeculectomy
Leukocyte DNA			
Cont(4)	F	63	None
Primary Cell Lines			
Cont(5)	M	10	None
Cont(6)	F	75	None
FECD(4)	M	71	FECD
FECD(5)	M	75	FECD
FECD(6)	F	90	FECD
FECD(7)	F	63	FECD
FECD(8)	F	70	FECD
FECD(9)	F	70	FECD
FECD(10)	M	59	FECD
FECD(11)	F	63	FECD

Short tandem repeat analysis was initially used to determine *TCF4* TNR expansion length in leukocyte DNA for FECD(1–3) and Cont(1–3). While this method is valuable for quantitation of medium sized repeat lengths (<200), it is not accurate for larger repeat sizes (>200). Since we hypothesized the corneal endothelium would contain larger expansions than found in leukocytes, we utilized Iso-Seq to assess *TCF4* transcript repeat length. The average number of circular consensus sequence (CCS) reads was slightly higher in control (4.4 million average) than FECD (3.5 million average) samples, but the number of mapped unique loci was similar between groups with 21,702 ± 2103 (mean ± standard deviation) from controls and 21,901 ± 2973 (mean ± standard deviation) from FECD. Additional output data from the Iso-Seq sequencing can be found in S1 Table.

### Characterization of *TCF4* CTG repeat length in leukocyte DNA

The CTG repeat length as measured by short tandem repeat assay in leukocyte DNA is listed in Table 2. The three FECD subjects were heterozygous for repeat expansion with a long allele ranging from 67–90 repeats. By definition, two of the three control subjects [Cont(1–3)] were also heterozygous for non-expanded allele lengths ranging from 12–28 repeats.

**Table 2:** Summary of repeat length in *TCF4* in leukocytes and corneal endothelium.

Sample	Leukocyte DNA—Number of repeats (short allele / long allele)	RNA in corneal endothelium					
		Sense reads				Antisense reads	
		Expanded allele		Non-expanded allele			
		Number of reads	Number of repeats	Number of reads	Number of repeats	Number of reads	Number of repeats
			Median (range)		Median (range)		Median (range)
**FECD(1)**	12 / 90	38	1601 (435–2787)	9	13 (12–21)	3	1263 (912–1589)
**FECD(2)**	15 / 78	21	1121 (210–2023)	0	-	0	-
**FECD(3)**	13 / 67	10	908 (320–2284)	1	12 (12)	1	12 (12)
**Cont(1)**	18 / 18	0	-	5	18 (17–18)	0	-
**Cont(2)**	12 / 18	0	-	0	-	0	-
**Cont(3)-1** *	12	0	-	8	12 (12–14)	0	-
**Cont(3)-2** *	28	-	-	1	29 (29)	-	-

*Cont(3)-1 and Cont(3)-2 refer to reads from each allele.

### Assessment of repeat size in RNA transcripts from corneal endothelial surgical specimens

**Fig 1:**
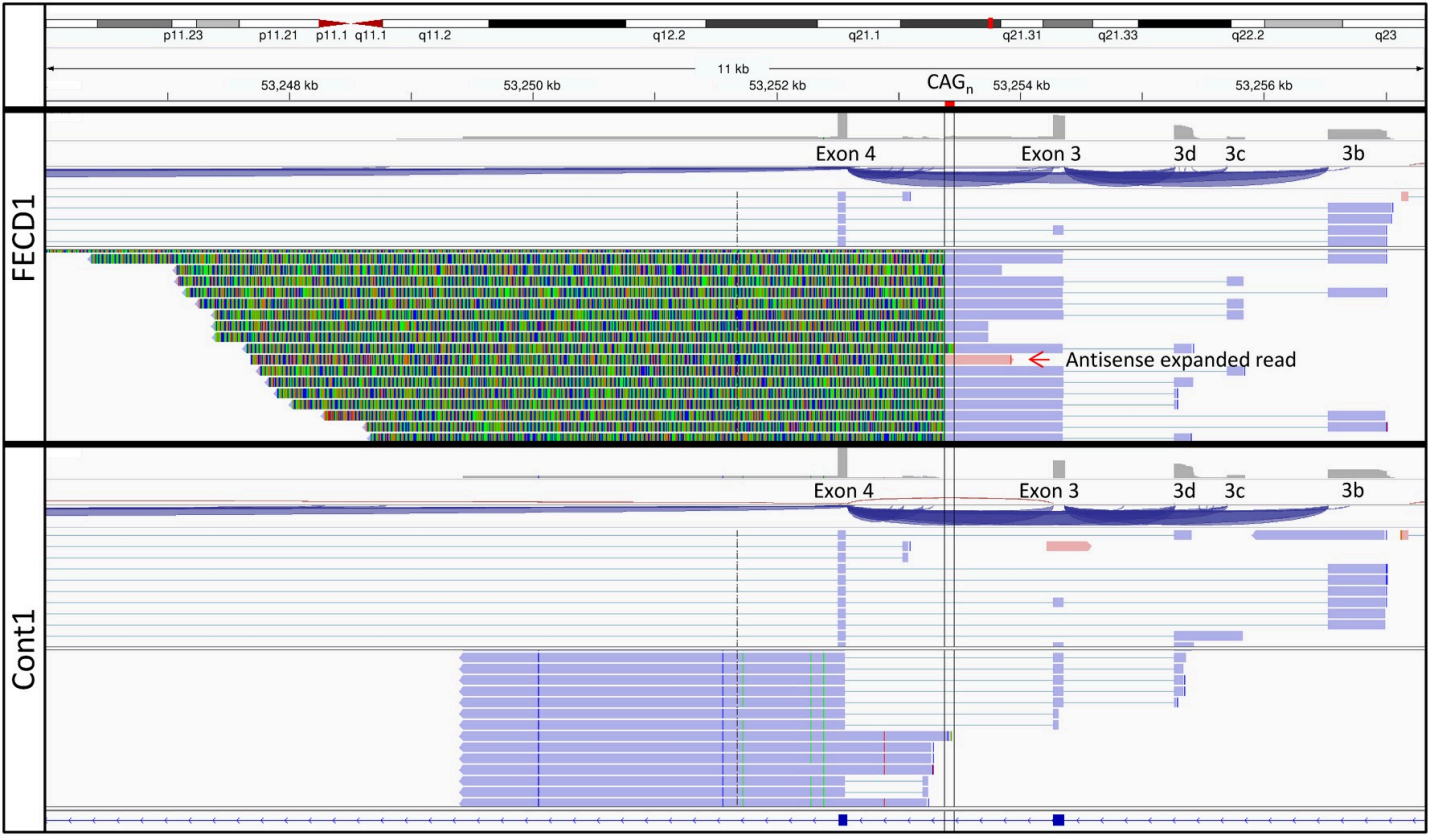
Iso-Seq reads from the corneal endothelium include repeat expansions greater than 6 kb in length. Iso-Seq reads spanning 11 kb from the IGV of FECD(1) (top two rows) and Cont(1) (bottom two rows). Locations of the CAG repeats (CAG_n_) and exons in this region as defined previously [12] are labelled. Each sample is shown twice to show the structure and diversity of different read types. The blue-green rich regions in the second FECD row are CAG expansions.

**Fig 2:**
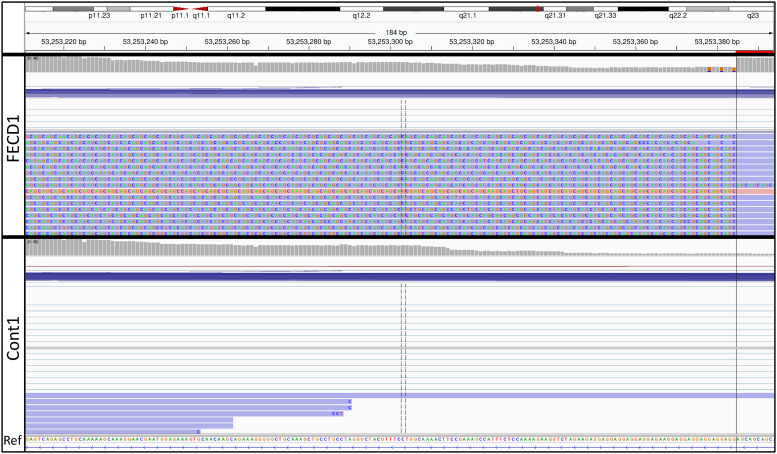
Representative assessment of CAG expanded repeats. Magnified view of the same samples as shown in Fig 1, showing the reference sequence (Ref) and the actual CAG sequence of the expanded repeats in FECD(1). The single antisense read in this view is shown with the pink background.

**Fig 3:**
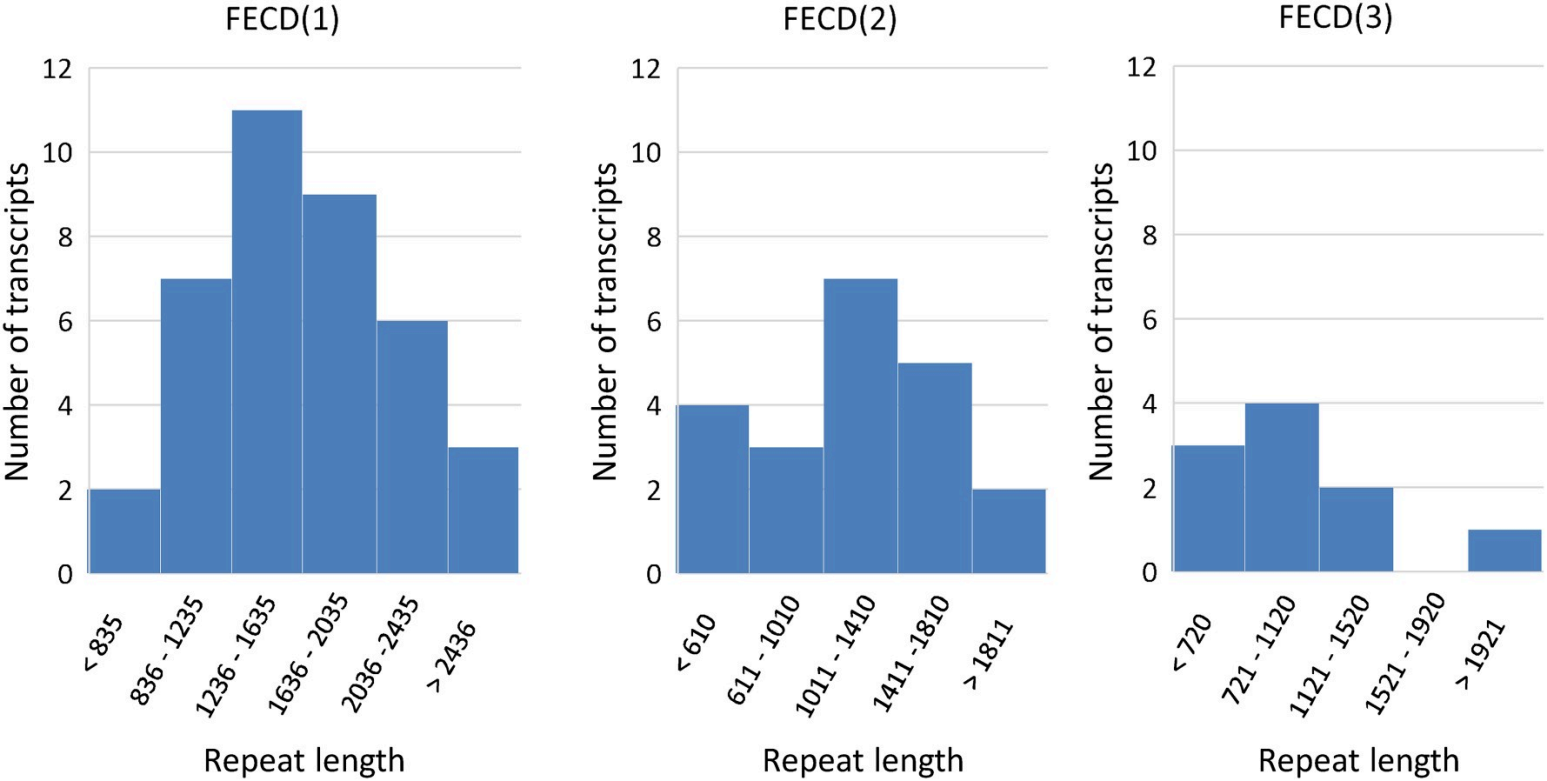
Repeat length size distribution of TCF4 transcripts originating from FECD expanded alleles. Various repeat lengths were identified in transcripts originating from each FECD patients expanded allele. Note that the shortest expanded repeat reads from Iso-Seq were 435 [FECD(1)], 210 [FECD(2)], and 320 [FECD(3)], which are >2.5-fold larger than TNR length in leukocyte DNA.

Visualization of the Iso-Seq unfiltered FLNC *TCF4* CCS reads in the Integrative Genomics Viewer (IGV) revealed CAG (CAG in RNA, CTG in DNA) repeat lengths as long as 6 kb (2000 CAG repeats) in all three FECD patients (Figs 1 and 2, representative samples). For each FECD sample, each of the expanded alleles showed a distribution of repeat lengths (Fig 3). The shortest expanded repeat reads from Iso-Seq for these samples were 435 [FECD(1)], 210 [FECD(2)], and 320 [FECD(3)] repeats. For all FECD samples, most of the Iso-Seq reads from expanded alleles terminated within the CAG repeats, so the size distribution of expanded repeats shown in Fig 3 are likely smaller than the full size of the repeats in these samples. Iso-Seq reads from the unexpanded alleles in these samples included reads containing the short CAG repeat in FECD(1) and FECD(3). FECD(3) also had an antisense read that correlated with the unexpanded CAG repeat on the sense strand (Table 2).

In addition to the sense reads, antisense reads containing expanded repeat sequence were identified in FECD(1) and FECD(3) (Table 2). FECD(1) had three antisense reads, all including expanded repeats with a mean size of 1263 repeats. Iso-Seq reads from the unexpanded alleles in FECD corneal endothelial samples included reads containing the CAG tract in FECD(1) and FECD(3) and one unexpanded antisense read in FECD(3) (Table 2).

The large repeat sizes in FECD corneal endothelial tissue originating from the *TCF4* alleles with TNR expansion contrasted with the *TCF4* alleles from control corneal endothelial samples, in that no reads were identified containing expanded repeats. The median size of the repeat reads in the control corneal endothelial samples [Cont(1) and Cont(3)] were within one repeat of the size measured by short tandem repeat analysis in leukocyte DNA samples.

### *TCF4* TNR expansion in primary HCECs

**Fig 4:**
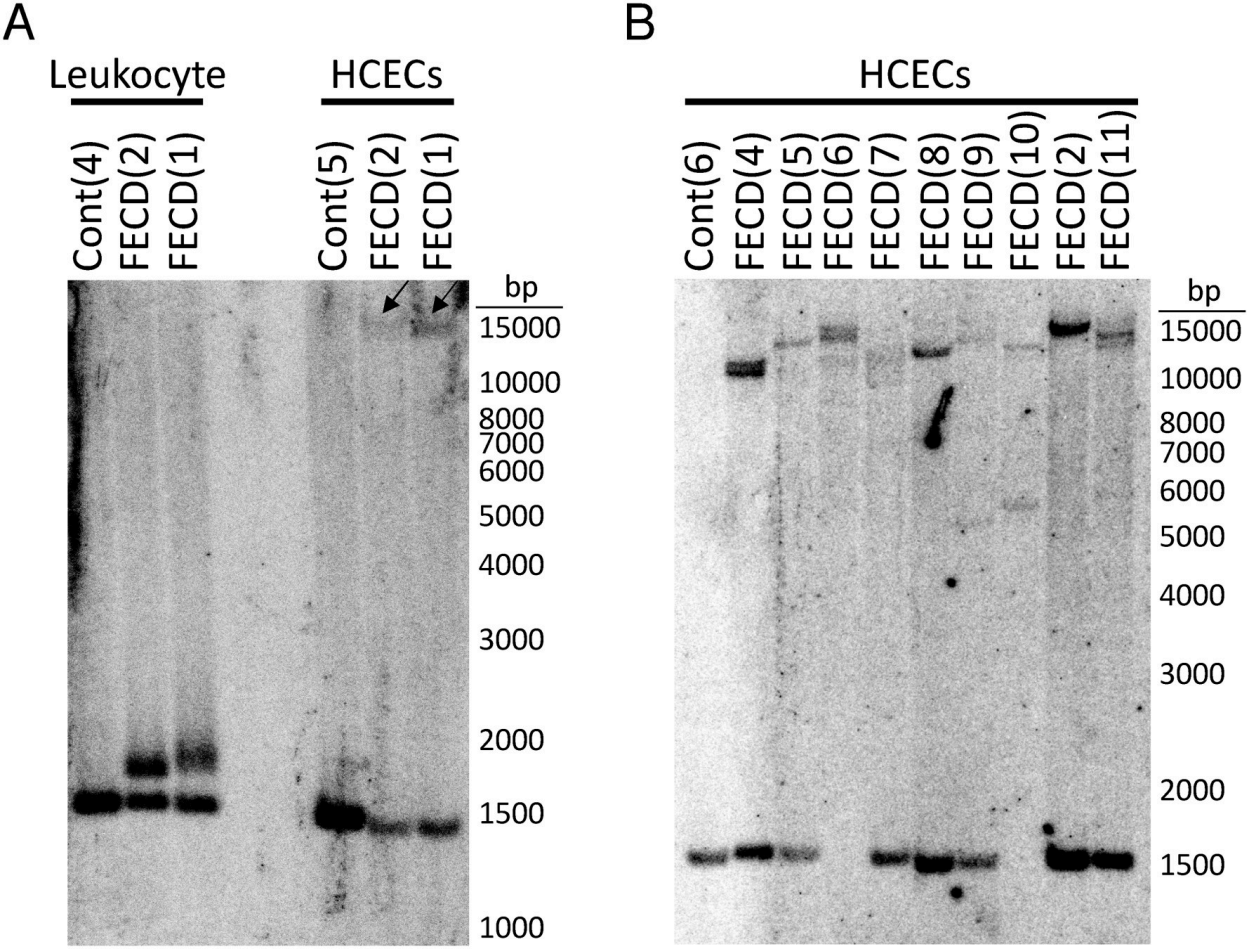
Southern blot of peripheral blood leukocyte and primary HCEC lines established from control and FECD patients. (A) *TCF4* repeats in DNA isolated from peripheral blood leukocytes (Lanes 1–3) and primary HCECs (Lanes 4–6). Lanes 1 [Cont(4)] and 4 [Cont(5)] represent samples obtained from different control patients. Lanes 2, 5 [FECD(2)] and 3, 6 [FECD(1)] are samples from two FECD patients with expanded repeats. Arrows indicate large molecular weight bands in FECD1 and FECD2 corresponding to approximately 15,000 base pairs and approximately 4500 repeats. For analysis, 5 μg leukocyte DNA and 20 μg primary HCEC DNA was digested with EcoRI and separated on an 0.8% agarose gel. (B) Southern blot of control and FECD primary HCEC cell lines showing large molecular weight bands ranging from approximately 5500—>15,000 base pairs. This corresponds to all FECD samples having at least one allele with a *TCF4* expansion of >1100 repeats. Genotype of *TCF4* expansion size as determined in leukocytes for each FECD HCEC line is as follows: FECD(4)—16/69; FECD(5)—25/71; FECD(6)—73/82; FECD(7)—18/52; FECD(8)—12/64; FECD(9)—12/66; FECD(10)—100/123; and FECD(11)—17/98. Note that FECD(2) was utilized as a positive control in both (A) and (B). For analysis, 5 μg of primary HCEC DNA was digested with EcoRI and separated on an 0.8% agarose gel.

For FECD(1) and FECD(2), the corneal endothelial sample obtained from the contralateral eye following endothelial keratoplasty was used to establish a primary cell line. Results from Southern blotting of DNA isolated from these patients are shown in Fig 4a. As described previously, hybridization with a TCF4 probe to EcoRI digested DNA from individuals lacking a repeat expansion in *TCF4* yields a single band with a size of approximately 1500 bp [Cont(4)], corresponding to repeat sizes of 12–20 repeats [10]. In individuals with long repeat expansions, higher molecular weight bands are detected, reflecting the size of expanded alleles in the source tissue. For FECD(1) and FECD(2) (lanes 3 and 2 respectively), expanded alleles (90 and 78 repeats) and non-expanded alleles (12 and 15 repeats) were identified in leukocyte DNA, agreeing well with other short tandem repeat analysis results (Table 2). However, DNA isolated from passage 2 of the primary cell lines established from the contralateral corneal endothelium of these patients [FECD(1) and FECD(2)] and a control corneal endothelial primary cell line [Cont(5)] revealed corresponding bands for unexpanded alleles (12–20 repeats) but no bands at the size of the expanded alleles identified from leukocyte DNA. Instead, FECD(1) and FECD(2) have bands that are approximately 15 kb, consistent with expansions of approximately 4500 repeats. These data are consistent with the large size of the Iso-Seq corneal endothelial transcripts found in RNA samples from the same patient.

To determine if this is a characteristic of FECD HCECs, we isolated genomic DNA from 8 additional primary HCEC lines we had previously established from FECD patients and assessed *TCF4* repeat length by Southern blot (Fig 4b). In all FECD HCEC primary cell lines, at least one allele contained a *TCF4* expansion that contained greater than 1100 repeats, consistent with our findings obtained from Iso-Seq transcript analysis and Southern blot analysis in FECD(1) and FECD(2). These additional primary HCEC cell lines showed expansions that were >10-fold longer than their corresponding TCF4 expansion repeat identified in leukocytes. It is interesting to note that in FECD(6) and FECD(10), both alleles showed expansion but to different degrees, consistent with their homozygous expansion observed in leukocytes [FECD(6)– 73/82; FECD(10)– 100/123].

### Altered pattern of protein isoforms produced in FECD

Transcription of human *TCF4* has been shown to utilize at least 18 different promoters [12, 13]. The long read sequencing of mRNA from our corneal endothelial samples confirmed that promoters 3b, 3c and 3d are used (promoters upstream of the intron containing the CTG repeat), with similar numbers of transcripts mapping to all three in both FECD and control samples. The long read sequencing data also showed that the upstream portion of the intron containing the CAG repeats is preferentially retained in FECD samples compared to control samples, consistent with our earlier observation [2]. It is notable that in the FECD samples this intron segment is retained in a portion of the transcripts that do not contain expanded repeats as well as in those that do contain repeat expansions. Only one of the three control samples [Cont(1)] produced any reads that mapped to this portion of intron 3 that is immediately upstream of the CAG repeats.

As shown in Table 3, more than 90% of the transcripts identified from corneal endothelium code for 5 of the 18 TCF4 protein isoforms (TCF4-A, B, C, D, and I) [12, 13]. In 5 of the 6 samples we sequenced, the most abundant isoforms were those that are unique to TCF4-A. This isoform was the most abundant in all three of the FECD samples (44–75% of all transcripts) and was also the most abundant form found in two of the three control samples [48–57%; Cont(1) and Cont(3)]. In Cont(2), the TCF4-A isoform was also abundant (18%) but the TCF4-B and TCF4-C isoforms each represented 30% of the total number of transcripts. The proportion of the 5 major isoforms were similar in FECD and control samples, with the only exception being TCF4-C, which showed a lower percentage of transcripts in FECD (5–9%) than in controls (10–30%).

**Table 3:** TCF4 promoter usage.

		A family		B family		C family		D family		I family		
Sample	Total TCF4 CCS Reads	CCS Reads	% total	CCS Reads	% total	CCS Reads	% total	CCS Reads	% total	CCS Reads	% total	% represented
FECD(1)	790	437	55.3	197	24.9	64	8.1	41	5.2	30	3.8	97.3
FECD(2)	467	204	43.7	124	26.6	43	9.2	43	9.2	16	3.4	92.1
FECD(3)	384	286	74.5	29	7.6	20	5.2	28	7.3	11	2.9	97.4
Cont(1)	1212	577	47.6	255	21.0	169	13.9	56	4.6	69	5.7	92.9
Cont(2)	207	38	18.4	63	30.4	62	30.0	10	4.8	0	0.0	83.6
Cont(3)	542	311	57.4	63	11.6	54	10.0	35	6.5	56	10.3	95.8

CCS–circular consensus sequence.

## Discussion

Approximately 78% of FECD patients in the United States have an expansion of a CTG repeat sequence in an intron of the *TCF4* gene. Traditionally, researchers in the field have generated estimations of repeat length from leukocyte DNA rather than the targeted diseased tissue and have correlated these data with mechanistic changes in affected corneal tissue which includes the role of RNA toxicity, repeat-associated non-AUG translation, and changes in TCF4 expression (see review [15]). In FECD, technical limitations have previously made it impossible to quantify the *TCF4* TNR expansion length in corneal endothelial DNA. Short read sequencing is incapable of estimating the actual size of expanded repeats beyond 200 repeats because reads consisting entirely of CAG repeats cannot be unambiguously mapped to a particular site in the genome. Southern blotting, as we performed on primary HCEC lines (Fig 4), is not possible with surgically-obtained corneal endothelial tissue samples because it requires more DNA than can be extracted. To circumvent these issues, we utilized Iso-Seq and found that the *TCF4* TNR was significantly longer in corneal endothelium when compared to the *TCF4* repeat length in peripheral blood leukocytes from the same individuals.

Long read sequencing of RNA from the corneal endothelium of three FECD patients yielded reads containing CAG repeats that were as long as 8300 bp (~2750 repeats), more than 20 times longer than the *TCF4* expanded repeat measured in their leukocyte DNA. None of these expanded CCS reads completely spanned the CAG repeats, because they do not contain sequences from both 5’ and 3’ flanking regions of the repeats. Thus, the full extent of the sizes of these expansions is likely to be even longer. This is the first direct confirmation of the transcription of these expanded repeats (including a limited number of antisense reads as previously noted by Hu et al. [16]) in cells from the corneal endothelium. Furthermore, the observation that transcripts containing the repeats are accumulating in corneal endothelial cells is interesting simply from the perspective that most primary transcripts containing intron sequences are effectively removed by the mRNA splicing machinery and degraded. Consequently, transcripts containing normal intronic sequences do not accumulate in appreciable amounts in the total RNA transcriptome. This likely accounts for the low total number of transcripts containing repeat sequences in the control samples.

Our findings indicate that the transcripts with the longest median size [1601 repeats in FECD(1)] actually yielded the most reads (38) while the expansion-containing transcripts with the smallest median size [908 in FECD(3)] yielded the fewest reads (10). This raises the possibility that the accumulation of these transcripts may be directly related to the length of the repeat sequence. We also note that the sample with the longest repeat expansions identified by Iso-Seq also had the longest repeat size in leukocyte DNA. These data suggest that the repeat lengths in DNA from the corneal endothelium are much longer than those measured in leukocyte DNA. This hypothesis is bolstered by direct measurement by Southern analysis of the repeat length in FECD patients [FECD(1) and FECD(2)] whose contralateral corneal endothelium was used to establish primary HCECs. Both of these samples had bands consistent with having 4000–5000 repeats, much longer than have been seen in any DNA samples from leukocytes.

One of the unanswered questions associated with FECD has been why patients with TNR expansions in the *TCF4* gene develop disease only in the corneal endothelium. The unique opportunity to collect blood and fellow-eye corneal endothelium (one used for Iso-Seq and the other for primary HCEC line generation) from the same patients, and to then determine that TNR expansion in the *TCF4* gene is longer in corneal endothelium DNA compared to those measured in leukocyte DNA is highly significant. The identification of longer expanded repeats in the corneal endothelium might help to explain why a repeat expansion in the widely expressed *TCF4* gene preferentially leads to disease in the cornea. It is also interesting to speculate on the small minority of patients that have expanded repeats as measured in peripheral blood leukocytes but do not develop FECD, even at advanced age [17]. If shorter expansions are not as efficient at leading to the pathogenesis of disease, it is conceivable that these individuals may have shorter *TCF4* TNR expansions in their corneal endothelial cells. If this is true, it suggests that early detection of the disease along with therapies to reduce the *TCF4* TNR expansion length may be a viable treatment option for the disease. Further validation studies with a larger sample size will be required before *TCF4* TNR expansion length and disease pathogenesis can be correlated.

Another benefit of long read sequencing for the analysis of mRNAs is information on distribution of protein isoforms produced in FECD relative to controls. Timmusk and colleagues have performed extensive analysis of transcription start sites from a variety of human cells [12]. In the most recent study, they concluded that the presence of CTG expanded repeats in *TCF4* reduced transcription from promoters immediately downstream of the repeats [13]. Our data are consistent with this conclusion, since the proportion of total predicted TCF4 protein isoforms originating from just downstream of the repeats (5–9%) was less than that observed in controls (10–30%) (C family, Table 3, corresponding to Exon 4 in Fig 1).

One potential limitation to using long read sequencing to annotate TNR sequence length is that preparation of the Iso-Seq libraries involves multiple cycles of PCR. It remains possible that multiple amplification cycles may lead to erroneous expansion of the repeat sequence. We believe this to be highly unlikely as we did not observe any expanded repeat reads that were anywhere near the sizes measured with short tandem repeat analysis of DNA from leukocytes. Even the shortest expanded repeat reads we observed from our samples (435, 210, and 320 repeats in samples FECD(1), FECD(2), FECD(3) respectively) were more than 2.5 times the repeat lengths measured in leukocyte DNA (90, 78, and 67 repeats in FECD(1), FECD(2), FECD(3) respectively). Additionally, we did not observe artefactual expansion of the unexpanded repeats in the control samples, and the observed sizes of the unexpanded alleles in the FECD patient samples were within one repeat of the sizes measured in short tandem repeat analysis of leukocyte DNA. Furthermore, multiple PCR cycles are also performed during the short tandem repeat analysis protocol, and no repeats of the sizes observed in the present Iso-Seq results were observed in those experiments or in Southern blots using DNA from leukocytes. One obvious control experiment to address this issue would be to correlate Iso-Seq-generated repeat length data in RNA with actual DNA repeat length in leukocytes. However, there is very little transcription from the upstream *TCF4* promoters in leukocytes, so this experiment would not yield usable information. This has been confirmed by analyzing Iso-Seq data on leukocyte RNA from other samples. We were able to generate corneal endothelial primary cell lines from the patient’s contralateral eye and using early passage cultures, demonstrated large DNA expansions consistent with the data obtained from the Iso-Seq studies.

Another limitation to this study is the small sample size. In searching our database of over 450 FECD patients with second eye surgery, we identified only 2 patients where we had established a primary cell culture from one corneal endothelial tissue, used the contralateral eye tissue for long-read sequencing, and were able to obtain a blood sample to isolate leukocyte DNA [FECD(1–2)]. With the continued development of long-read sequencing technology [18], future studies will be necessary to maximize its use in additional patients to confirm the longer *TCF4* TNR expansions in the corneal endothelium and its association with FECD.

In summary, data using Iso-Seq long-read sequencing of RNA isolated from FECD patients are consistent with the hypothesis that repeat expansions in the corneal endothelium are significantly longer than those assayed in their leukocyte DNA. As noted previously, tissue-specific differences in the size of repeat expansions have been described in other repeat expansion diseases and it has been hypothesized that the increased size of expansions in target tissues contributes to the pathogenesis and tissue specificity of disease [19]. Our findings of >4-fold and even up to 50-fold increased size of CAG repeats in RNA from corneal endothelium and in primary HCEC lines established from the same FECD patients suggests that a similar process exists in FECD. Further work with larger numbers of samples and other non-ocular tissues will be required to rigorously test this hypothesis and to evaluate other possible disease risks in patients with FECD.

## Supporting information

S1 Raw images (PDF)

S1 Table Iso-Seq data generated from FECD and control samples. (DOCX)
