# Supplementary figures and images for: Comparison of TCF4 repeat expansion length in corneal endothelium and leukocytes of patients with Fuchs endothelial corneal dystrophy
Source: PLoS One. 2021 Dec 2;16(12):e0260837. doi: 10.1371/journal.pone.0260837 (PMC8638873; doi:10.1371/journal.pone.0260837)

Raw image  
for Figure 3a

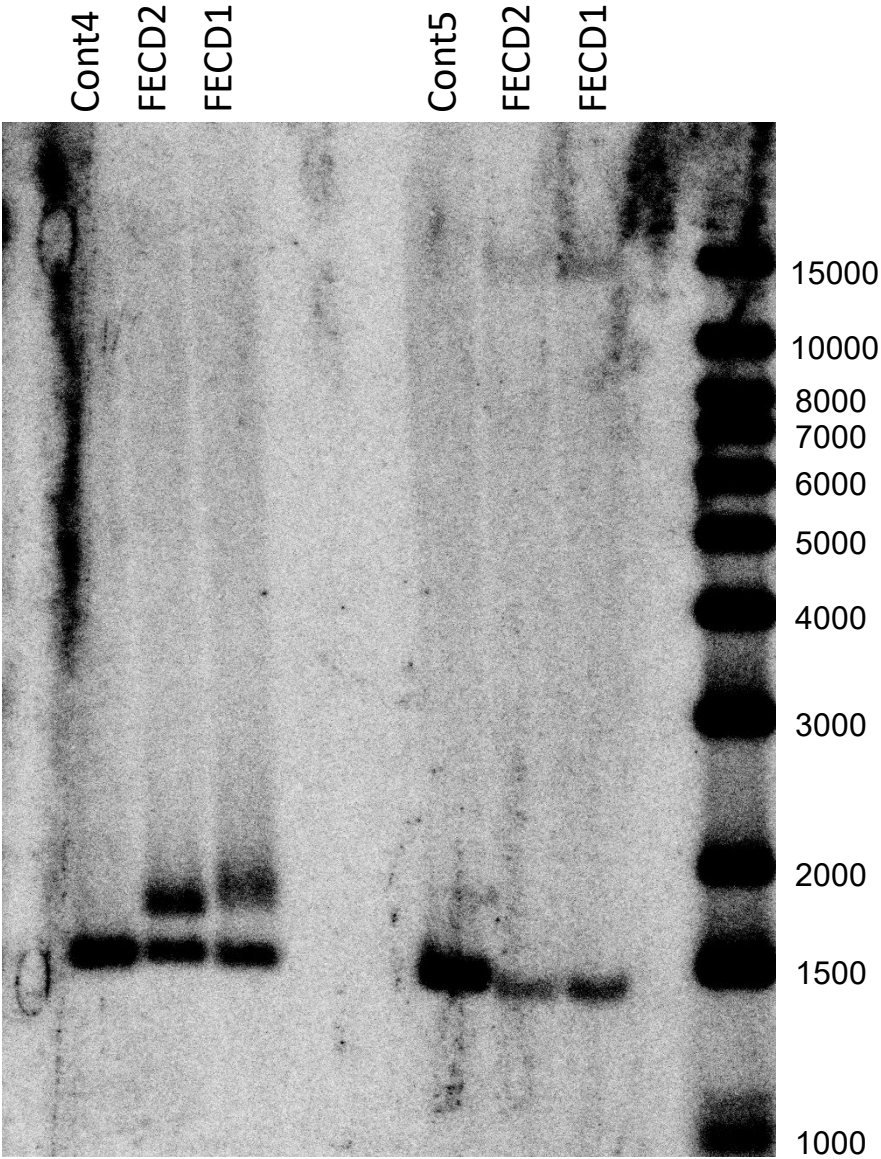

Raw image  
for Figure 3b

HCECs

Cont(6)

FECD(4)

FEC(5)

FEC D(6)

FEC(7)

FEC D(8)

FEC D(9)

FEC(10)

FEC(2)

FEC(11)

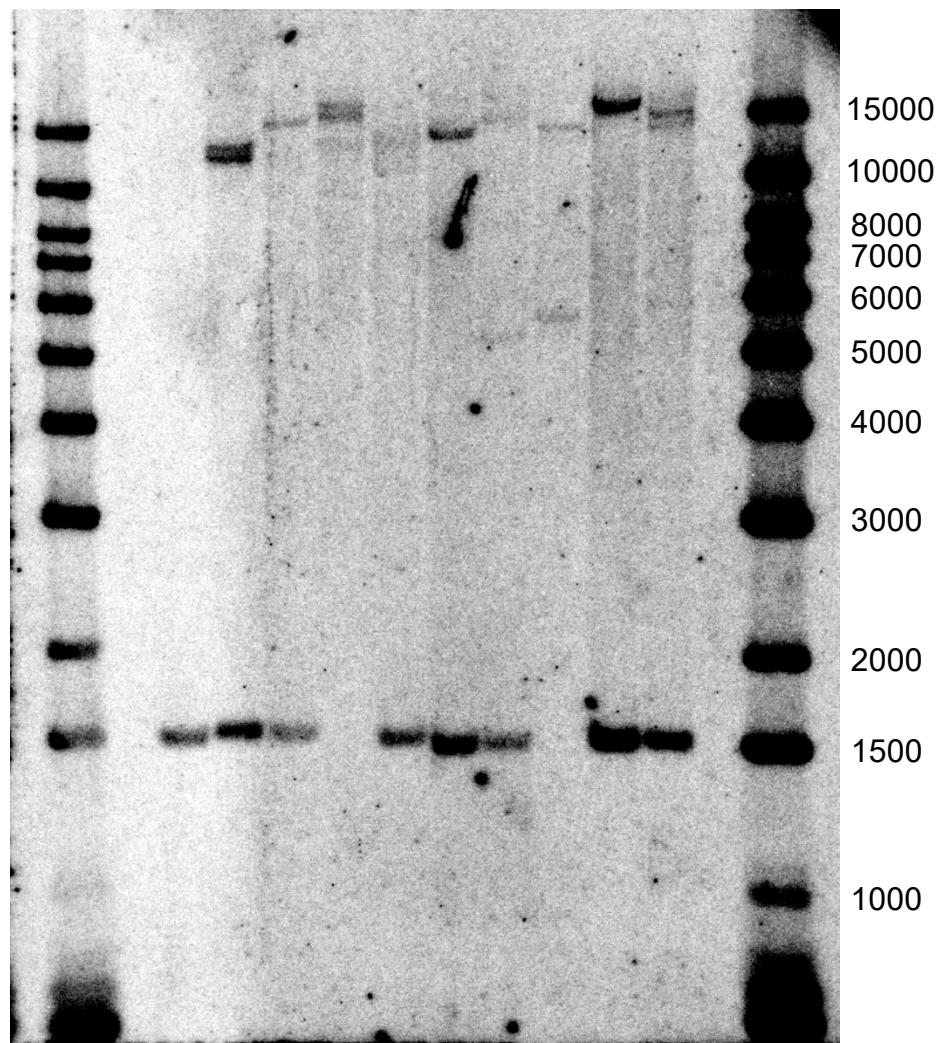

Supplement: S1 Raw images — (PDF) [file pone.0260837.s001.pdf]
