# Supplementary material for: Comparison of TCF4 repeat expansion length in corneal endothelium and leukocytes of patients with Fuchs endothelial corneal dystrophy
Source: PLoS One. 2021 Dec 2;16(12):e0260837. doi: 10.1371/journal.pone.0260837 (PMC8638873; doi:10.1371/journal.pone.0260837)
Supplement: S1 Table — (DOCX) [file pone.0260837.s002.docx]

**S1 Table. Iso-Seq data generated from FECD and control samples.**

| **Sample** | **RIN** | **Number of CCS reads** | **CCS bases** | **Number of mapped unique loci** |
| --- | --- | --- | --- | --- |
| **FECD(1)**  **Cont(1)** | 8.7  9.5 | 4,629,020  4,549,284 | 19,068,766,226  19,278,022,657 | 20497  20923 |
| **FECD(2)**  **Cont(2)** | 7.5  8.0 | 2,713,818  3,839,952 | 10,304,604,092  14,795,202,396 | 25316  20100 |
| **FECD(3)**  **Cont(3)** | 8.7  8.9 | 3,102,888  4,674,401 | 8,657,225,085  14,840,286,989 | 19889  24084 |

RIN – RNA integrity number

CCS – circular consensus sequence
